# 

**Published:** 1856-12

**Authors:** 


### Notices of Exchanges.

*The Ohio Medical and Surgical Journal.* Edited by John Dawson, M. D. Professor of Anatomy and Physiology in Starling Medical College, assisted by R. Grundy, M. D. Published bi-monthly, at $2,00 per annum.

The September number of this journal is filled, in the main, with important and interesting matter. Its Review department is extensive and discriminating. Its senior editor we have known from our early boyhood, and we are pleased to see the journal giving evidence of that industry and thought for which he was ever noted.

*The Peninsular Journal of Medicine and the Collateral Sciences.* Edited by Zina Pitcher, M. D., &c., A. B. Palmer, M. D., &c., assisted by W. Brodie, M. D. and E. P. Christian, A. M., M. D.

The Peninsular Journal is issued monthly at $2,00 per annum. Its contents consist of "Original Communications," "Selections," "Editorial, and Book notices," and "Miscellaneous." Its matter is interesting and important to the medical reader, and would be profitable to the dentist.

*Charleston Medical Journal and Review.* C. Happoldt, M. D. Editor and publisher. Published bi-monthly, at $4,00 per annnm.

The Journal and Review makes a fine appearance. Each number is embellished with a fine steel plate engraving of a prominent medical man, living or dead. The September number contains a likeness of the late Dr. Chapman of Philadelphia, and the usual amount of original and select matter. Though we cannot take time to read all our exchanges, it is one we do not slight thus far.

---

*The St. Louis Medical and Surgical Journal.* Edited by M. L. Linton, M. D. and W. M. McPheeters, M. D. Issued bi-monthly at $2,00 per annum.

This is a handsome journal of about a hundred pages. Its contents are arranged under the heads of "Original Communications," "Reviews and Bibliographical Notices," "Record of Medical Science" and "Editorial." The September number is before us, and contains a great variety of interesting matter.

---

*The Boston Medical and Surgical Journal.*

This old *week*ly shows, as yet, no signs of *weak*ness. No. 11 vol 55 is before us. That it has sustained itself so long is evidence both of its vitality and worth. Being a weekly it is able to give the latest professional news usually in advance of its competitors. Our medical readers all know it; and the same is true of many dentists. Will the dental profession ever sustain a weekly journal?

---

*The Memphis Medical Recorder.* Published every two months by the Memphis Medical College. Edited by A. P. Merrill, M. D., &c.

The September number contains an article on infant mortality, a variety of bibliographical notices, selections, editorial, miscellanies, &c. Much of its matter is interesting and important to the physician, and, as the editor is "assisted by his colleagues," it is fair to infer that the "Recorder" is a faithful exponent of the views and interests of the Memphis Medical College.

---

*The Western Lancet.* A monthly journal of practical medicine and Surgery. Edited by T. Wood, M. D. and Geo. C. Blackman, M. D.

The "Lancet" is just approaching the close of its seventeenth volume. It is an old acquaintance of the Medical Faculty, and should be familiar to the dental profession. The October issue is an interesting number, and contains some pungent "reviews and notices;" and not the least *caustic* of its articles is the one on the "Ohio State Medical Society" by "Paul Caustic" himself. We regard this *epistle of Paul* as adapted to other atmospheres than that of the state society. Perhaps he would lend some of his suggestions to the dental profession.

We learn with regret, that Dr. Wood is about to retire from the chair editorial; but we console ourselves that the "Lancet" is to be left in such good keeping. Dr. Blackman is "tried," and as the duties of his chair are less laborious than Dr. Wood's, it is possible he may have more time to devote to the "Lancet," than it was possible for Dr. Wood to give it.

The "Lancet" is printed by Messrs. Wrightson & Co. and the style of execution is like that of the "Register," which, we flatter ourselves is as neat as anything extant.

---

*The Cincinnati Medical Observer.* Edited by George Mendenhall, M. D. Professor of Obstetrics in Miami Medical College, John A. Murphy, M. D., Professor of Materia Medica in Miami Medical College and Edward B. Stevens, M. D.

Many of the readers of the "Register" will at once recognize, in the senior editor our former professor of Pathology in the "Ohio College of Dental Surgery." To these the "Observer" needs no recommendation. It is sufficiently endorsed. The October number is filled with quite a variety of matter, both original and select.

The Observer is published monthly, at $2,00 per annum, by E. B. Stevens, M. D.

---

*The Inventor.*

This is a neat monthly of 32 pages. To those who wish to keep posted up on inventions, we can freely say it is the work they want. Its illustrations are decidedly neat, and it contains much information of general interest. It contains official lists of the Patents granted at Washington, with their claims, and notices of the most valuable new inventions for which patents have been issued. We read the "Inventor" with interest, and sometimes with amusement. A New York correspondent in the October number in a "History of Fire Engines and Utensils" announces the astonishing fact that "In England steam power has been applied with success" to fire engines. The editor had better apply cold water to that writer's head, and send him to Cincinnati, to see "Old Joe," "The Citizen's Gift," and their comrades *squirt.* Then he *can* write about *Fire* engines. Applied with success in England, indeed! Why, here, if a fire bursts out in a hay-loft; it is expected that, not only the building, but *part of the hay*, will be saved.

The "Inventor" is published by Low, Haskel, & Co., 304 Broadway, New York.

---

*Our Exchanges.*

We intended to notice in this number, all our medical exchanges; but, before we got throngh, an attack of old fashioned ague seized us, and we laid aside the pen. If we thought the ague was brought on by this *tampering with medicine*, we would certainly consider ourselves excused from saying anything farther on the subject of medical journals, especially if they emanate from malarious districts. But, "*Post hoc non ergo propter hoc.*"

### Editorial.

### THE OHIO COLLEGE OF DENTAL SURGERY.

Many inquiries are made by members of the Profession, evincing a desire to know definitely the condition of the college, and the course of instruction given in it.

For the gratification of such, we would state briefly, that the college property is owned by a joint stock association of dentists, including a few who are directly interested in the cause of dental science, but not practical dentists. This association meets annually at the close of the college session to receive the report of the faculty, and to transact such other business as may be called for. At each meeting an "Examining Committee" composed of three dentists and two physicians, is elected for the ensuing year, which committee meets with the faculty, and assists in conducting the examinations of the candidates, each member having equal power with a member of the faculty, in passing or rejecting those examined. The association arranges the course of instruction, and elects the professors of the several departments. In short the association is the living principle of the institution—is the institution itself. The board of trustees possesses the *legal* power of the institution; but when the society was organized, the board transferred all the active duties to it, believing that its members were better acquainted with the wants of the profession than those of the board could be. Accordingly the trustees merely ratify the actions of the association to give them legal force.

The college edifice was built with direct reference to dental instruction. A correct front view of it is given on the cover of our Annual Announcements. It is four stories high, and consists of twelve rooms. Two of these are large lecture rooms, one in the second, and the other in the third story, each calculated to accommodate one hundred students or upwards, The laboratory is the same size as the lecture rooms. In the fourth story are three dissecting rooms, one large and two small ones. The remaining rooms are devoted to the infirmary department, museum, cabinets, faculty room, &c.

The course of instruction can, perhaps be better understood by inserting the daily order of lectures, than by any other method:

"DAILY ORDER OF LECTURES."

| | M | T | W | T | F | S |
|---|---|---|---|---|---|---|
| Dr. Taylor | 8 | 8 | | 8 | 8 | |
| " Chapman | 10 | 10 | 10 | 10 | 10 | |
| " Taft | 9 | 9 | 8 & 11 | | | |
| " J. B. Smith | 11 | 11 | | 11 | 11 | |
| " Watt | | | 9 | 9 | 9 | 9 |
| " H. R. Smith | 2 | 2 | 2 | 2 | 2 | 2 |
| CLINICS. | | | | | | |
| " Taft | 2 | 2 | 2 | | | |
| " Taylor | | | | | | 8 |

It will be found by analyzing the above table, that the students are constantly engaged from eight to twelve o'clock, each day of the week, except saturday. On this day their morning duties close at ten—allowing them a respite till two, p. m.

By the system of classification adopted, the clinics of Dr. Taft, at 2 p.m. do not, in the least, interfere with the demonstrations of Dr. Smith at the same hour.

We have endeavored, with minuteness and brevity, to answer at least some of the many questions propounded to us, in reference to these things. We wish all to understand exactly the true state of affairs. When the profession know just what is being done, they are the better prepared to advise in regard to what should be done, and also better qualified to assist in doing it. W

### MISSISSIPPI VALLEY ASSOCIATION.

The M. V. Association will commence its next meeting at the Dental College on Wednesday, February 25th, 1857, at 10 o'clock, A. M. The subjects proposed for discussion, by the Executive Committee, will be seen in the present number. We hope members will endeavor to adhere as closely as possible to the questions under consideration, as a full report of the discussions will be published.

### OUR LAST NUMBER.

Our readers, no doubt, noticed that the October number of the Register was much above its ordinary size, but does any one think it was too large? Would you not all like to see it that size, or a little larger, each issue? We know very well *we* would; and we believe *you* would. Now we cannot make it that size, ourselves; but *you* can. And we'll tell you how. By each one of you feeling it to be his own special and personal duty to extend our circulation a little, the thing will be accomplished like magic. We would not speak so plainly if we expected to make a pecuniary profit from the Register, but, as soon as we have prompt paying subscribers sufficient at all to justify, we are resolved to enlarge the Register at once, to one hundred and sixty pages of reading matter. Will you help us?

### IN ADVANCE.

Subscribers will please bear in mind that our terms require payment *in advance.* In order to succeed in our effort, advance pay is required; and you will read with a better relish when the Register is your own. The present number brings us to the middle of the year, and yet we have received on subscription less than half the expenses of a single issue. As the printer must be paid, we send bills with the present number, hoping the response will promptly relieve us from all embarrassment. Money may be sent by mail at our risk.

### OHIO DENTAL COLLEGE ASSOCIATION.

This association stands adjourned to meet at the College on Monday, February 23d, 1857. A full attendance and punctuality are earnestly solicited. The commencement exercises of the College will take place on Wednesday evening, February 25th, 1857.

---

☞ The Dental News Letter devotes one page of its advertising sheet to "*nostrums*." We thought the dental journals had all got "one peg above" such things. The M. D. editor most likely, had nothing to do with it. Afraid it was you, Mc. Was it?

---

☞ The March number of the Register will be delayed a little, to obtain the proceedings of the College, and Mississippi Valley Associations, and the commencement exercises.

---

☞ We again call the attention of those who receive the Register, to the fact that if it is not *returned* or *refused* at the post office, we will consider them subscribers, and act accordingly.

---

☞ We call special attention to the advertisement of Messrs. Thorne & Co.

An extensive assortment of dental goods, in almost every variety, may always be found at their store, and also everything in the *fancy* line that the most fastidious taste could desire.

### MEDICAL COLLEGE OF OHIO.

### SESSION OF 1856-'57.

### The Thirty-Seventh Annual Course of Lectures

In this Institution, will commence on the 15th day of October, and continue four months.

### FACULTY.

**L. M. LAWSON, M. D.,**
*Professor of the Theory and Practice of Medicine.*

**GEORGE C. BLACKMAN, M. D.,**
*Professor of the Principles and Practice of Surgery and Clinical Surgery.*

**THOMAS WOOD, M. D.,**
*Professor of Anatomy.*

**N. T. MARSHALL, M.D.,**
*Professor of Obstetrics and Diseases of Women and Children.*

**JAMES GRAHAM, M. D.,**
*Professor of Materia Medica and Therapeutics.*

**JOHN H. TATE, M. D.,**
*Professor of Physiology, Hygiene, and Medical Jurisprudence.*

**JOHN A. WARDER, M. D.,**
*Professor of Chemistry and Toxicology.*

**E. S. WAYNE,**
*Chemical Assistant and Lecturer on Practical Pharmacy.*

**S. G. ARMOR, M. D.,**
*Professor of Pathology and Clinical Medicine.*

**R. L. REA, M. D.,**

*Demonstrator of Anatomy.*

Fees for the whole Course, $72; Matriculation (paid once only,) $5; Hospital, $5; Graduation, $25; Demonstrator's Ticket, $5; Board, $2,50 to $3 a week.

A Preliminary Course, free to all Students, will commence on the 1st of October, and continue until the commencement of the regular Session.

Clinical Instruction by the Professors of Clinical Medicine and Surgery, will be given daily at the Commercial Hospital.

Students may rely upon an abundant supply of *material.*

Letters addressed to the Dean, or any member of the Faculty, will be promptly answered.

Students, on arriving in the city, can obtain further information by calling on the Dean or Janitor at the College, on Sixth street, between Race and Vine.

S. G. ARMOR, M. D.,
87 Seventh Street,
**Dean of the Faculty.**

THOMAS WOOD, M. D.,
*Registrar*

### BALTIMORE COLLEGE OF DENTAL SURGERY.

**SIXTEENTH ANNUAL SESSION, 1856-57.**

**FACULTY.**

CHAPIN A. HARRIS, D. D. S., M. D.
*Principles of Dental Surgery.*

THOMAS E. BOND, M. D.
*Therapeutics and Materia Medica.*

WASHINGTON R. HANDY, M. D.
*Anatomy and Physiology.*

PHILIP H. AUSTEN, D. D. S., M. D.
*Mechanical Dentistry.*

EDWARD MAYNARD, D. D. S., M. D.
*Theory and Practice of Dental Surgery.*

REGINALD N. WRIGHT, M. D.
*Chemistry and Metallurgy.*

CHRISTOPHER JOHNSTON, M. D.
*Microscopical and Comparative Anatomy of the teeth.*

GEORGE W. NEIDICH D. D. S.
*Demonstrator of Dental Surgery.*

The Seventeenth Annual Session of the Baltimore College of Dental Surgery, will open on the first of October, and close on the third of March. The Regular Course of Lectures will commence on the third of November.

The month of October will be devoted to Practical Dentistry and Anatomical Dissections.

The large practice of the Infirmary offers to the Student superior facilities for the acquirement of a *practical* knowledge of Medical, Surgical, and Mechanical Dentistry.

Professors' Tickets, $110; Infirmary Ticket, $10; Dissection Ticket, (optional) $10; Matriculation Fee, $5; Diploma Fee, $30.

Good Board may be procured at from $3 50 to $4 0[illegible] per week. For further information address.

P. H. AUSTEN, *Dean of the Faculty.*

*No.* 76 *Sharp Street.*

---

### DENTAL DEPOT.

### JOHN KLEIN,

MANUFACTURER OF

### PORCELAIN TEETH,

### Gold & Tin Foil, Gold & Silver Plate, Wire, Spiral Springs

INSTRUMENTS, &c., &c.

*No.* 16½ *North Eighth street, Philadelphia, Pa.*

Constantly on hand a general assortment of Improved Plate, Pivot, Molar, Bicuspids, and Gum Teeth, at reduced prices; together with an assortment of Dental Instruments, Materials, Tools. Lathes, Wheels, Molding Flasks, Gold and Tin Foils, Files, Blow Pipes, Lamps, Head Rests, &c., &c. All Orders promptly filled.

### THE OHIO COLLEGE OF DENTAL SURGERY.

### Session of 1856-7.

---

The regular course of lectures in this institution, commences on the first Monday of November, and closes on Tuesday, succeeding the fourth Monday of February.

### FACULTY.

JAMES TAYLOR, M. D., D. D. S.,
*Professor of the Institutes of Dental Science.*

C. B. CHAPMAN, M. D.,
*Professor of Anatomy and Physiology.*

J. B. SMITH, M. D.,
*Professor of Pathology and Theraputics.*

JONATHAN TAFT, D. D. S.,
*Professor of Operative Dentistry.*

H. R. SMITH, M. D., D D.. S.,
*Professor of Mechanical Dentistry.*

GEORGE WATT, D. D. S., M. D.
*Professor of Chemistry and Metallurgy.*

The Infirmary will be kept open during the summer and during the month of October special attention will be paid to operations before such of the class as can come in by that time. Those desiring *extra* opportunities for practice should avail themselves of this month's privileges.

To meet the wants of those engaged in practice, half course tickets are issued, thus enabling them to make out a full course of graduation without leaving their homes and practice for one entire session.

Students should bring with them such instruments and text books as they may have on hand.

Candidates for graduation must have attended two full courses of Lectures, the last in this school. Certificates of four years Dental Practice, or of one full course in any respectable Dental or regular Medical College, will be received as equivalent to the first course.

---

### TERMS OF ADMISSION.

The tickets are considered as cash, and when not paid for in advance, a note to the Faculty, with approved security will be required.

| | |
|---|---|
| Tickets for the entire course, - - - - - - - | $100 00 |
| Matriculation Ticket, - - - - - - | 5 00 |
| Diploma Fee, - - - - - - | 25 00 |

Good and respectable board can be had from $2 50 to $4 per week.

GEORGE WATT, Dean.

TENTH VOLUME

OF THE

### DENTAL REGISTER OF THE WEST.

EDITED BY

J. TAFT and G. WATT.

TERMS: - - - - - - - - - $3 PER ANNUM, IN ADVANCE.

**Advertisements Inserted as Follows:**

One page, per annum........................................................$25 00
One-half page, per annum.................................................. 15 00
Quarter page, per annum.................................................. 10 00
Card (five lines or less),................................................... 5 00,

Shorter periods than one year, by special arrangement.

☞ Contributions to the pages of the *Register* respectfully solicited.

Exchanges and correspondents will please address, "Dental Register," or "Taft & Watt, Cincinnati, Ohio."

